# ROS-mediated autophagy increases intracellular iron levels and ferroptosis by ferritin and transferrin receptor regulation

**DOI:** 10.1038/s41419-019-2064-5

**Published:** 2019-10-28

**Authors:** Eunhee Park, Su Wol Chung

**Affiliations:** School of Biological Sciences, College of Natural Sciences, University of Ulsan, 93 Daehak-ro, Nam-gu, Ulsan, 44610 South Korea

**Keywords:** Apoptosis, Autophagy

## Abstract

Ferroptosis is a novel form of programmed cell death in which the accumulation of intracellular iron promotes lipid peroxidation, leading to cell death. Recently, the induction of autophagy has been suggested during ferroptosis. However, this relationship between autophagy and ferroptosis is still controversial and the autophagy-inducing mediator remains unknown. In this study, we confirmed that autophagy is indeed induced by the ferroptosis inducer erastin. Furthermore, we show that autophagy leads to iron-dependent ferroptosis by degradation of ferritin and induction of transferrin receptor 1 (TfR1) expression, using wild-type and autophagy-deficient cells, BECN1^+/−^ and LC3B^−/−^. Consistently, autophagy deficiency caused depletion of intracellular iron and reduced lipid peroxidation, resulting in cell survival during erastin-induced ferroptosis. We further identified that autophagy was triggered by erastin-induced reactive oxygen species (ROS) in ferroptosis. These data provide evidence that ROS-induced autophagy is a key regulator of ferritin degradation and TfR1 expression during ferroptosis. Our study thus contributes toward our understanding of the ferroptotic processes and also helps resolve some of the controversies associated with this phenomenon.

## Introduction

Iron is essential for the survival of nearly all organisms, as it serves as a cofactor for a host of biochemical processes, including oxygen storage, oxidative phosphorylation, and enzymatic reactions required for cellular proliferation^1^. However, the levels of free iron in a cell must be tightly regulated to avoid the generation of reactive oxygen species (ROS) via the Fenton reaction^2^. Transferrin receptor 1 (TfR1) and ferritins are well-known regulators of cellular iron. TfR1 is a transmembrane glycoprotein responsible for internalizing the transferrin-bound iron, which is then released into the cytoplasm and stored in a non-toxic form inside metalloprotein complexes called ferritins^3^. Ferritin is a complex of 24 subunits, consisting of two basic subunits: a heavy subunit (FTH1) and a light subunit (FTL).

Autophagy is the natural, regulated, destructive biological process that disassembles unnecessary or dysfunctional components of a cell^4^. The core machinery of autophagy consists of over 30 autophagy-related proteins, including BECN1 and LC3B. During autophagy, double-membrane vesicles are formed that engulf the damaged proteins and organelles and fuse with lysosomal vesicles^5^. BECN1 is an autophagosome initiation protein and LC3B is a structural component of the autophagosomes^6^. Autophagy is generally a stress-responsive survival mechanism. However, a controversial theory is that autophagy may also be a cell death mechanism i.e., autophagic cell death^7,8^. Recent studies have shown that autophagy degrades the iron-storage macromolecule ferritin, termed as ferritinophagy^9^. Degradation of ferritin increases the cellular iron levels, leading to accumulation of ROS and ultimately cell death.

Ferroptosis is a novel form of regulated cell death, characterized by an iron-dependent increase in lipid peroxidation. However, this process has not yet been well-defined in a chronological manner^10,11^. Erastin is a small molecule that is capable of triggering ferroptosis^11^. The ferroptotic cell death triggered by erastin is significantly inhibited by antioxidants (e.g., α-tocopherol, butylated hydroxytoluene, and β-carotene) and iron chelators (e.g., deferoxamine), suggesting that ROS- and iron-dependent signaling is required for erastin-induced ferroptosis^11^. According to the original study by the Stockwell group in 2012, erastin-induced ferroptosis in fibrosarcoma cells lacks the morphological and biochemical characteristics of apoptosis, necrosis, and autophagy^11^. However, recent studies have suggested that genetic inhibition of the autophagy pathway regulates the process and function of ferroptosis^12–14^. Whether ferroptosis and autophagy-induced cell death are dependent upon each other is currently controversial and not well understood. In this study, we have investigated a direct correlation between ferroptosis and autophagy using wild-type and autophagy-deficient fibroblastic cells. We found that erastin-induced ferroptotic cell death and intracellular iron levels are depleted in autophagy-deficient cells. Interestingly, erastin-enhanced early ROS seems to induce the autophagy process in fibroblastic cells.

## Results

### Erastin induces autophagy-dependent cell death

**Fig. 1:**
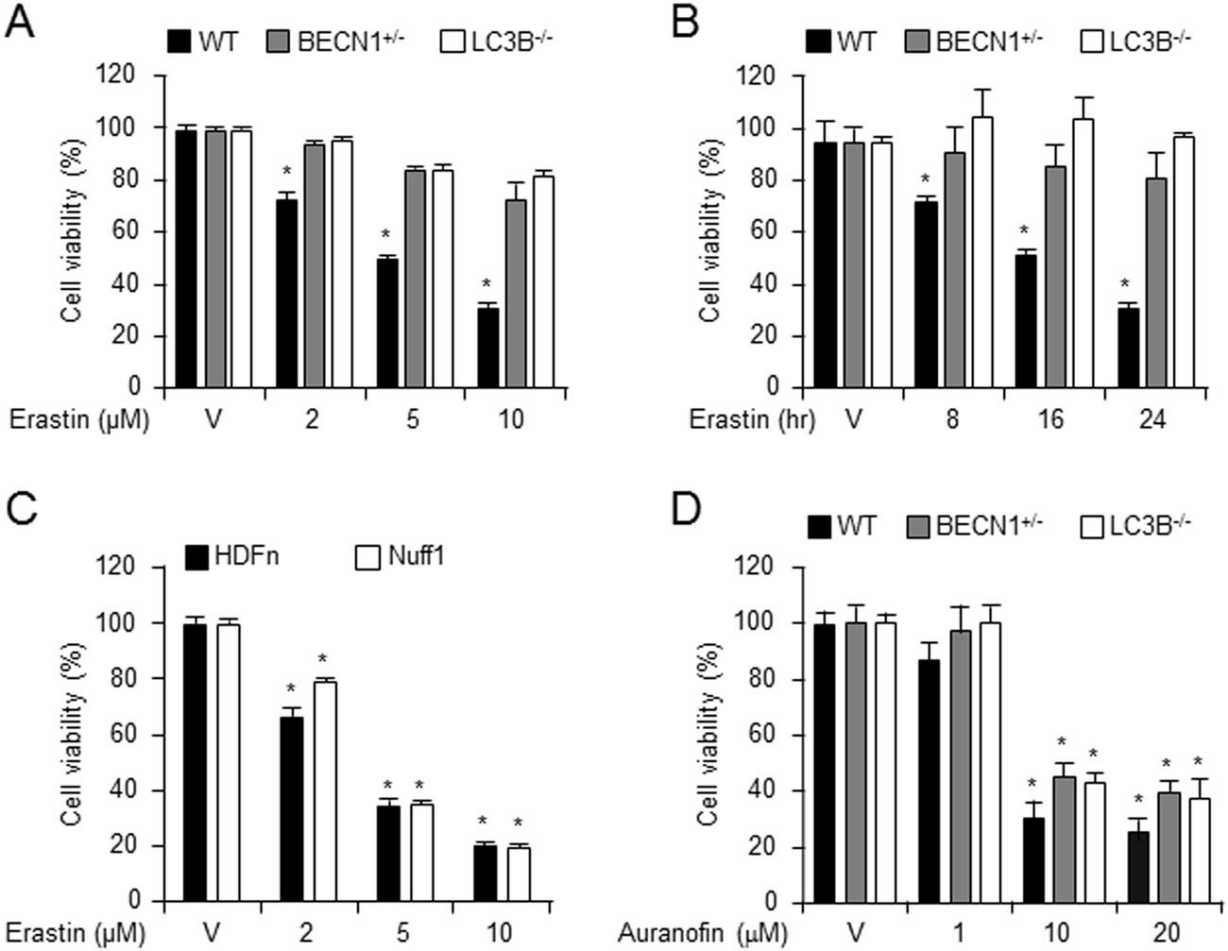
Erastin-induced ferroptotic cell death was decreased in autophagy-deficient cells. Cell viability assay was performed using wild type (n = 12), BECN1^+/−^ (*n* = 12), and LC3B^−/−^ (*n* = 12), primary mouse fibroblastic cells treated with indicated doses of erastin for 24 h (**a**) or treated for different time durations with 5 μM erastin (**b**). Cell viability was investigated in primary human dermal neonatal fibroblast (HDFn) and foreskin adult fibroblast (NuFF) following treatment with erastin (24 h) at the indicated concentration (**c**). Cell viability was investigated in wild type (*n* = 12), BECN1^+/−^ (*n* = 12), and LC3B^−/−^ (*n* = 12), primary mouse fibroblastic cells following treatment with indicated doses of auranofin for 24 h (**d**). All data shown are the mean ± SD from three independent experiments. *P < 0.05 indicates significant decrease compared with vehicle

**Fig. 2:**
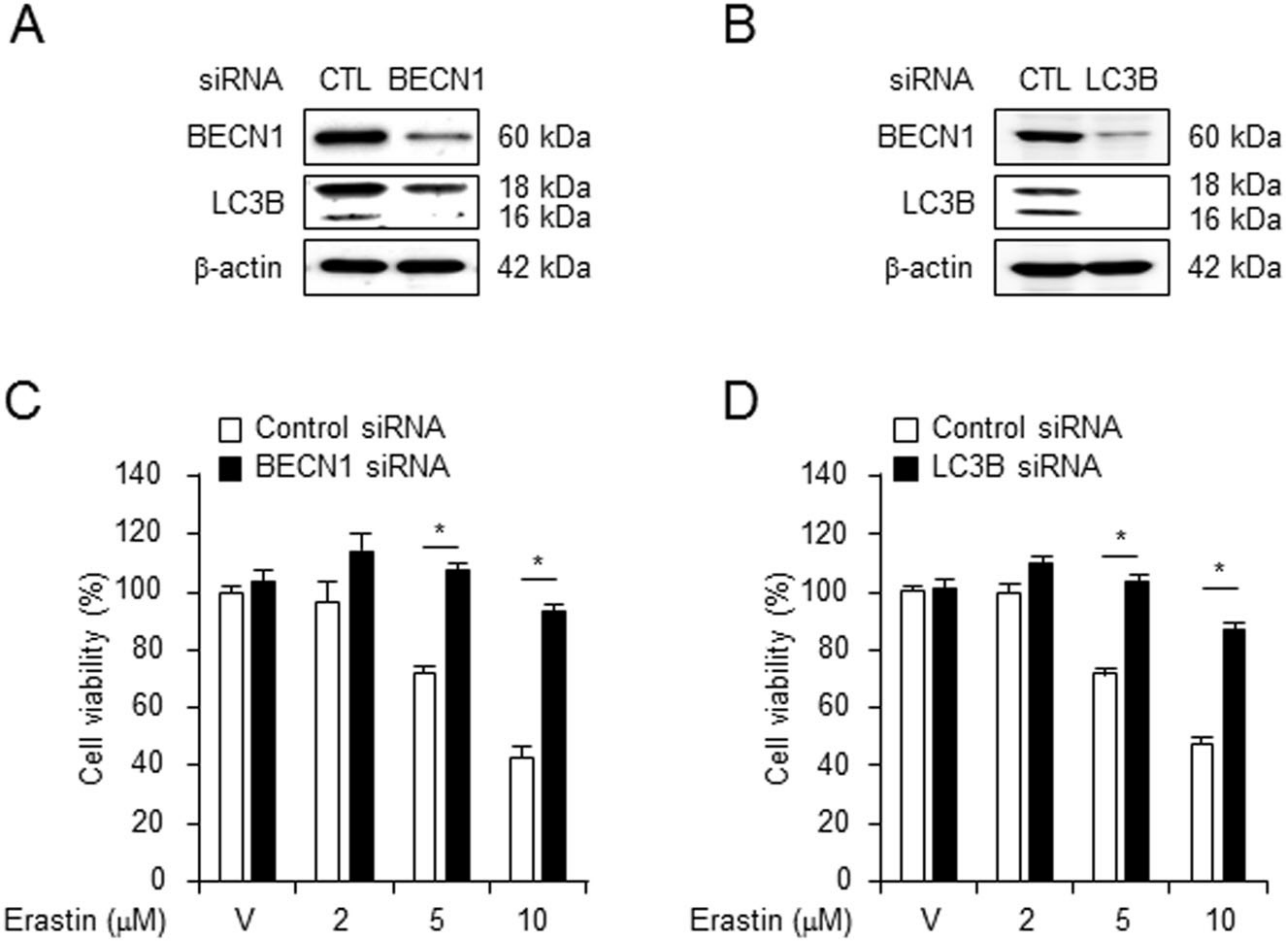
Autophagy-dependent ferroptosis was induced by erastin. Wild-type fibroblastic cells were transfected with control, BECN1, or LC3B siRNA and western blot was performed to verify the downregulation of BECN1 or LC3B expression (**a**, **b**, respectively). β-actin was used as loading control. Cell viability was measured in control (*n* = 3), BECN1 (*n* = 3), or LC3B (*n* = 3) siRNA transfected cells 24 h after vehicle or different doses of erastin treatment (**c**, **d**). All data shown are the mean ± SD from three independent experiments. *P *<* 0.05 indicates significant increase compared with control siRNA

Ferroptosis is a newly discovered iron-dependent process of cell death^11^. To investigate the role of autophagy in erastin-induced ferroptotic cell death, we harvested lung fibroblastic cells from BECN1^+/−^, LC3B^−/−^, and wild-type mice and characterized the primary lung fibroblast cell using rapamycin treatment (Supplementary Fig. 1). The BECN1^+/−^, LC3B^−/−^, and wild-type fibroblasts were treated with different concentrations of vehicle (control) or erastin for different time durations, and the cell viability was measured. The viability of BECN1^+/−^ and LC3B^−/−^ fibroblastic cells was enhanced in presence of 2 μM erastin (93.14% and 94.86%, respectively) and 10 μM erastin (72.01% and 81.14%, respectively), compared with that of wild-type cells treated with 2 μM (72.29%) or 10 μM (30.86%) erastin (Fig. 1a) and were similar results using propidium iodide (PI) staining (Supplementary Fig. 2). Further, the cell viability decreased after 5 μM erastin treatment for 8 h (71.42%) and 24 h (30.57%) in wild-type cells, but not in BECN1^+/−^ and LC3B^−/−^ fibroblastic cells (Fig. 1b). To examine whether erastin could induce the cell death in other primary human dermal neonatal fibroblastic cells (HDFn) or foreskin adult fibroblastic cells (NuFF), we treated these cells with erastin and then performed a cell viability assay. Significant increases in cell death were observed in both human primary fibroblastic cells by WST-1 assay (Fig. 1c). No significant differences in cell death were observed between human and mouse cells. In addition, to ascertain whether the enhanced cell viability in BECN1^+/−^ and LC3B^−/−^ cells is specific to erastin treatment, we treated these and wild-type cells with the cell death inducer, auranofin. A similar level of cell death was observed in BECN1^+/−^, LC3B^−/−^, and wild-type fibroblastic cells 24 h after auranofin administration (Fig. 1d), suggesting that the effects were erastin-specific. Moreover, knockdown of BECN1 or LC3B by siRNA prevented erastin-induced cell death in wild-type fibroblastic cells (Fig. 2). These data together suggest that the erastin-induced cell death in primary fibroblastic cells is associated with the autophagy process.

### Inhibition of autophagy disrupts erastin-triggered cell death

**Fig. 3:**
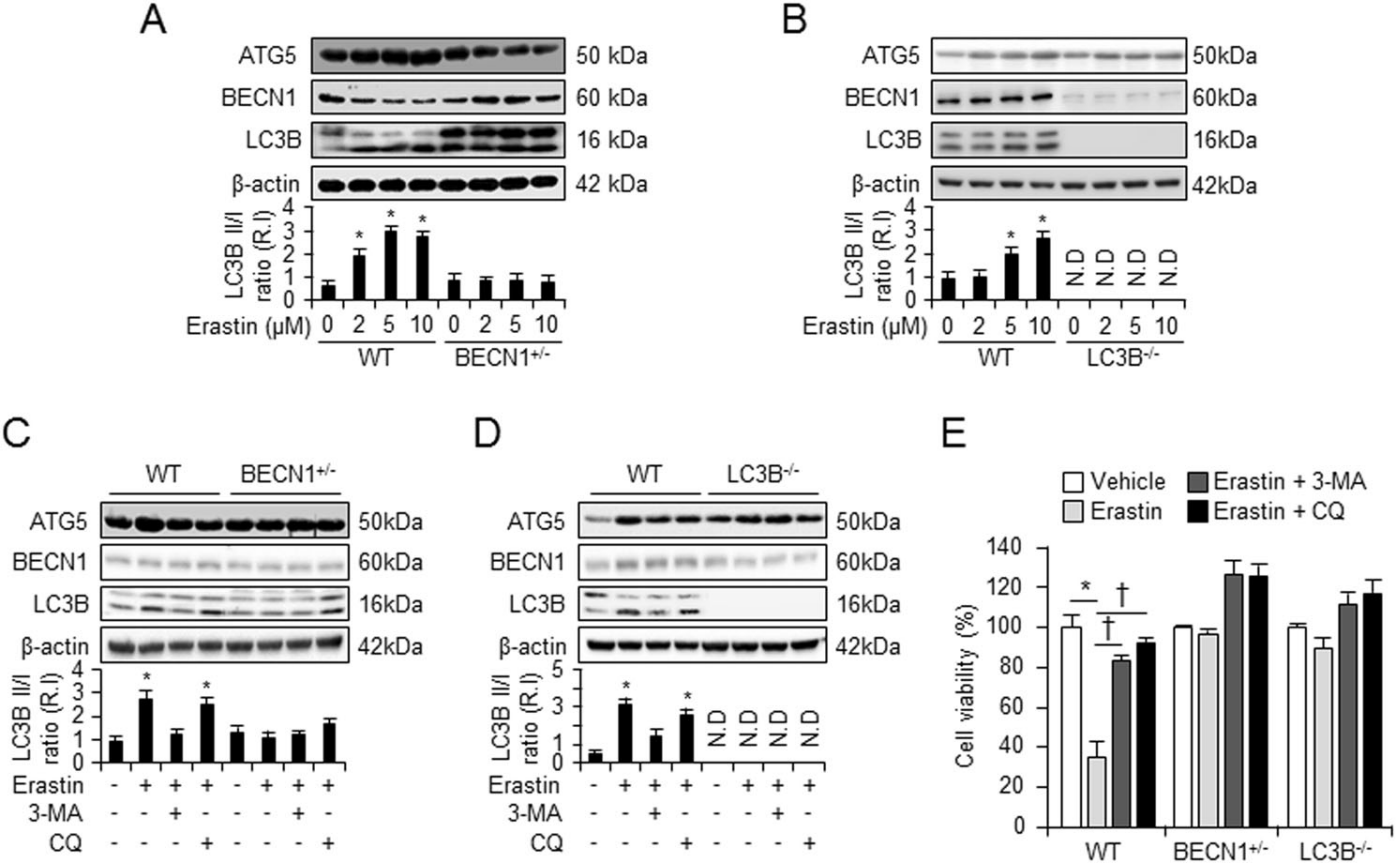
Inhibition of autophagy decreased erastin-induced ferroptotic cell death. Wild type (*n* = Wi3) or autophagy-deficient cells (BECN1^+/–^ (*n* = 3) or LC3B^−/−^ (*n* = 3)) were treated with various doses of erastin for 24 h and autophagy-related proteins, ATG5, BECN1, and LC3B, were analyzed by western blot (**a**, **b**). *P < 0.05 indicates significant increase compared with vehicle. Wild type or autophagy-deficient cells (BECN1^+/−^ (*n* = 3) or LC3B^−/−^ (*n* = 3)) were pretreated with autophagy inhibitors (3-MA or CQ) 1 h before erastin (5 μM) treatment for 24 h, and autophagy-related proteins, ATG5, BECN1, and LC3B, were analyzed by western blot (**c**, **d**). *P *<* 0.05 indicates significant increase compared with vehicle. Cell viability was assayed 24 h after vehicle, erastin, or erastin plus autophagy inhibitor (3-MA or CQ) treatment in wild type (*n* = 12), BECN1^+/−^ (*n* = 12), or LC3B^−/−^ (*n* = 12) (**e**). All data shown are the mean ± SD from three independent experiments. *P *<* 0.05 indicates significant decrease compared with vehicle; ^†^*P* *<* 0.05 indicates significant increase compared with erastin treatment

To verify whether erastin induces autophagy, we examined whether autophagy levels change in the presence of erastin in wild type, BECN1^+/−^, or LC3B^−/−^ fibroblastic cells. We treated these fibroblastic cells with vehicle or various doses of erastin (2, 5, or 10 μM) and harvested total protein 24 h after cell treatment. LC3B is a central protein in the autophagy pathway and is widely used as a biomarker of autophagosome^15^. In wild-type fibroblastic cells, the levels of LC3B-II (lower band of LC3B) protein was found to increase upon treatment with low dose of erastin (2 μM), and a more striking increase was evident at higher doses of erastin (5 and 10 μM), compared with treatment with vehicle (Fig. 3a, b). However, enhanced levels of LC3B-II were not observed in BECN1^+/−^ and LC3B^−/−^ fibroblastic cells after erastin treatment (Fig. 3a, b), suggesting that the erastin-induced LC3B-II expression is dependent on the autophagy process. To confirm whether increases in LC3B activation correlated with increased autophagic flux during the erastin treatment, we repeated these experiments by treating cells with erastin in the presence or absence of 3-methylaldehyde (3-MA), an inhibitor of phosphatidylinositol 3-kinase (PI3K) that blocks autophagosome formation, or chloroquine (CQ), an inhibitor of autophagosome-lysosome fusion. Treatment with 3-MA further decreased the activation of LC3B-II in vehicle or erastin-treated wild-type fibroblasts, indicative of autophagic activity (Fig. 3c). Consistently, CQ treatment further elevated the activation of LC3B-II in wild-type fibroblasts (Fig. 3c). In contrast, the erastin-treated BECN1^+/−^ and LC3B^−/−^ fibroblastic cells did not show decreased or elevated activation of LC3B-II upon 3-MA or CQ treatments, respectively (Fig. 3c, d). Next, to determine the role of autophagy in erastin-triggered cell death, we used a WST-1 assay to determine whether the autophagy inhibitors affected erastin-induced cell viability in wild type, BECN1^+/−^, and LC3B^−^^/−^ fibroblasts. As shown in Fig. 3e, in wild-type fibroblasts, the erastin-induced cell death recovered in the presence of autophagy inhibitors, 3-MA or CQ, compared with erastin treatment alone. However, similar treatment had no effect on BECN1^+/−^, and LC3B^−/−^ fibroblast cells (Fig. 3e). These data indicate that erastin-induced ferroptosis is an autophagy-dependent process and autophagy is a key activator of erastin-induced cell death.

### Depletion of autophagy attenuates lipid peroxidation in erastin-induced ferroptosis

**Fig. 4:**
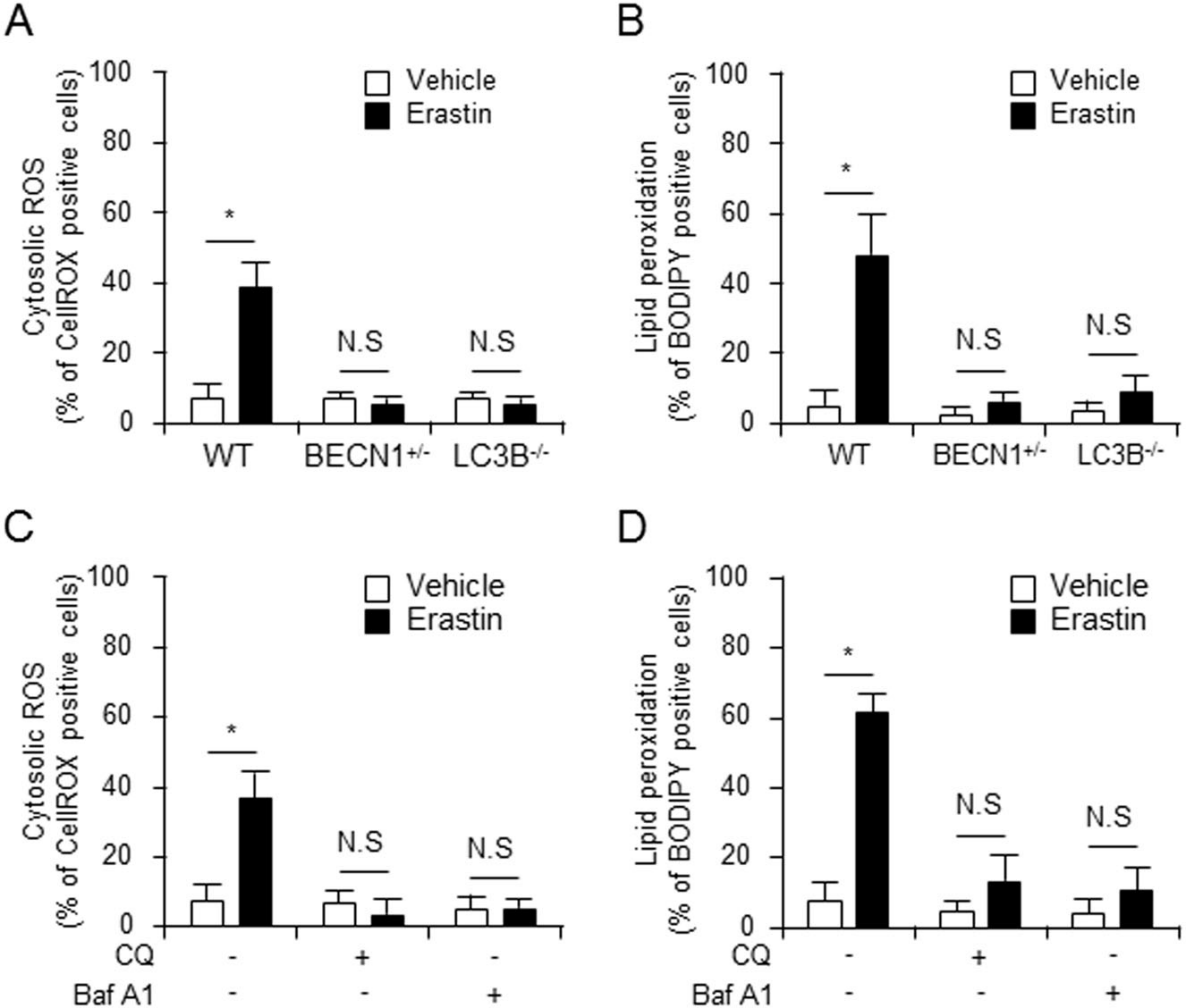
Erastin-induced lipid peroxidation was disrupted in autophagy-deficient cells. Wild type, BECN1^+/−^, or LC3B^−/−^ fibroblastic cells were treated with vehicle or erastin (5 μM) for 24 h. Cytosolic ROS (**a**) and lipid peroxidation (**b**) were assayed for by flow cytometry using the fluorescent probes CellROX® Deep Red (cytosolic ROS) and C11-BODIPY (lipid peroxidation), respectively. Wild-type fibroblastic cells were treated with vehicle, erastin (5 μM), or erastin plus autophagy inhibitor (CQ or Baf A1) for 24 h and cytosolic ROS and lipid peroxidation were measured (**c**, **d**). Fluorescence of each probe was measured using FlowJo software program. The mean percentages ± SD of positive cells per total cells are shown in plots. Values are mean ± SD, *n* = 6. *P *<* 0.05 indicates significant increase compared with vehicle; NS, not significant

ROS accumulation is one of the hallmarks of ferroptosis. Consistently, ferroptosis inhibitors (such as ferrostatin) and various antioxidants or ROS scavengers can all completely inhibit cellular ROS accumulation and ferroptotic cell death^16^. We, thus, hypothesized that depletion of autophagy protein levels such as BECN1 or LC3B may contribute to the regulation of erastin-induced ROS production and cell death in fibroblastic cells. To investigate the role of autophagy in lipid peroxidation, wild type, BECN1^+/−^, and LC3B^−/−^ fibroblastic cells were treated with vehicle or erastin and the cytosolic reactive oxygen species (ROS) (Fig. 4a) and lipid peroxidation (Fig. 4b) were assayed by flow cytometry using the fluorescent probes CellROX and C11-BODIPY, respectively. As shown in Fig. 4a, b, erastin treatment increased cytosolic ROS and lipid peroxidation (38.81% and 47.76%, respectively) compared with vehicle (6.97% and 4.72%, respectively) in wild-type fibroblastic cells, but not in BECN1^+/−^ and LC3B^−/−^ fibroblastic cells. To verify whether the erastin-induced lipid peroxidation is dependent on the autophagy pathway, wild-type fibroblastic cells were treated with autophagy inhibitors, CQ, or bafilomycin A1 (Baf A1), blocker of the fusion between autophagosomes and lysosomes, in the absence or presence of erastin. The erastin-induced cytosolic ROS and lipid peroxidation were disrupted upon inhibition of the autophagy process by the administration of CQ (3.51% and 13.25%, respectively) and Baf A1 (5.02% and 10.25%, respectively), compare with erastin treatment alone (36.75% and 61.25%, respectively) (Fig. 4c, d). These data suggest that autophagy might positively regulate erastin-triggered cell death by increasing lipid peroxidation.

### Autophagy deficiency inhibits erastin-induced intracellular levels of iron

**Fig. 5:**
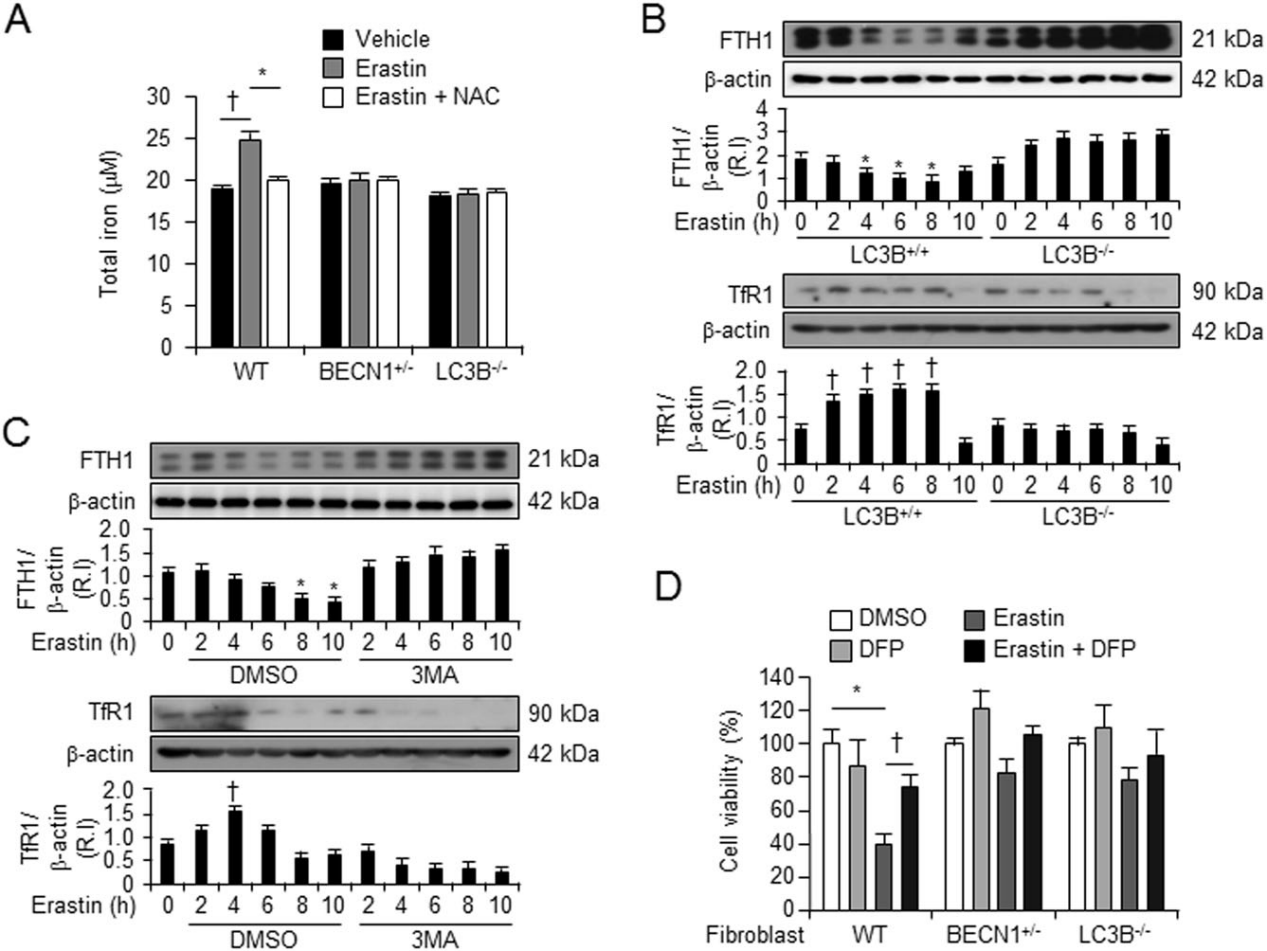
Levels of erastin-enhanced intracellular iron were restrained in autophagy-deficient cells. The wild type (*n* = 6), BECN1^+/−^ (*n* = 6), or LC3B^−/−^ (*n* = 6) fibroblastic cells were treated with vehicle or erastin (5 μM) in the absence or presence of the ROS scavenger, NAC, for 24 h and intracellular iron levels were assessed using a commercial assay (**a**). ^†^*P* *<* 0.05 indicates significant increase compared with vehicle. *P *<* 0.05 indicates significant decrease compared with erastin alone. The wild type, LC3B^+/+^ (*n* = 3), and LC3B^−/−^ (*n* = 3) fibroblastic cells were treated with vehicle or erastin (5 μM) for indicated time. Total protein was harvested and the protein levels of FTH1 and TfR1 were analyzed using western blot; β-actin was used as an internal loading control (**b**). *P *<* 0.05 indicates significant decrease compared with vehicle. ^†^*P* *<* 0.05 indicates significant increase compared with vehicle. The wild type fibroblastic cells were treated with vehicle or erastin (5 μM) in the absence or presence of autophagy inhibitor (*n* = 3), 3-MA (10 μM, *n* = 3)). Total protein was harvested and the protein levels of FTH1 and TfR1 were analyzed using western blot; β-actin was used as an internal loading control (**c**). *P *<* 0.05 indicates significant decrease compared with vehicle. ^†^*P* < 0.05 indicates significant increase compared with vehicle. These experiments were performed as three individual experiments and representative data is shown here. The wild type (*n* = 9), BECN1^+/−^ (*n* = 9), or LC3B^−/−^ (*n* = 9) fibroblastic cells were treated with vehicle, DFP (5 μM), or erastin (5 μM) in the absence or presence of DFP for 24 h and cell viability was assessed (**d**). All data shown are the mean ± SD. *P *<* 0.05 indicates significant decrease compared with vehicle; ^†^*P* *<* 0.05 indicates significant increase compared with erastin alone

Erastin-induced ferroptotic cell death involves the accumulation of intracellular iron, resulting in the production of cytosolic and lipid ROS^11^. We, therefore, examined the intracellular iron content in wild-type and autophagy-deficient cells upon various treatments. Intracellular levels of total iron were increased 24 h after erastin (5 μM) administration in wild-type fibroblastic cells, but not in BECN1^+/−^ and LC3B^−/−^ fibroblastic cells. Furthermore, erastin-induced intracellular iron in wild-type fibroblastic cells was found to decrease in the presence of the antioxidant, NAC (Fig. 5a). However, NAC treatment of BECN1^+/−^ and LC3B^−/−^ fibroblastic cells had no effect on the intracellular iron content (Fig. 5a). The intracellular levels of iron can increase upon degradation of iron protein complexes, such as ferritin, which forms a complex of 24 subunits consisting of a mixture of ferritin heavy (FTH) and light chains (FTL)^17^. Especially, ferritin is degraded via a recently identified autophagic process, ferritinophagy, and FTH1 is a substrate of ferritinophagy leading to lysosomal degradation^18,19^. Therefore, we determined whether low levels of intracellular iron in autophagy-deficient cells were related to autophagy-mediated degradation of FTH1 in the presence of erastin. We treated wild-type or autophagy-deficient LC3B^−/−^ fibroblastic cells with vehicle or erastin and harvested total protein 2, 4, 6, 8, or 10 h after treatment. The protein levels of FTH1 were found to decrease over time in wild-type fibroblastic cells in the presence of erastin (Fig. 5b). However, protein levels of FTH1 were enhanced in autophagy deficient, LC3B^−/−^, fibroblastic cells (Fig. 5b). To confirm that the degradation of ferritin occurred via the autophagy pathway, the cells were pretreated with the autophagy inhibitor 3-MA for 1 h before erastin treatment, and then the protein levels of FTH1 were determined. After erastin administration, protein levels of FTH1 decreased in wild-type fibroblastic cells, but increased in 3-MA-pretreated wild-type fibroblastic cells (Fig. 5c). The transferrin receptor (TfR1) is needed for the import of iron into the cell and is regulated in response to intracellular iron concentration^20^. Therefore, we analyzed the protein levels of TfR1 in autophagy-deficient cells (LC3B^−/−^) and autophagy inhibited cells (by inhibitor 3-MA), after erastin administration (Fig. 5b, c). The protein levels of TfR1 were increased after 6 h of erastin administration in wild-type fibroblastic cells (Fig. 5b, c). However, decreased levels of TfR1 were found 2 h after erastin administration in autophagy inhibitor (3MA)-treated wild-type fibroblastic cells (Fig. 5c). Interestingly, the protein levels of TfR1 were not increased in autophagy-deficient cells (LC3B^−/−^) after erastin administration (Fig. 5c). To verify whether autophagy is involved in iron-dependent cell death by erastin, we next observed the cell viability 24 h after erastin treatment in the absence or presence of iron chelator, deferiprone (DFP). Erastin-induced cell death recovered in the presence of iron chelator, DFP, in wild-type fibroblastic cells, but not in autophagy-deficient fibroblastic cells and DFP recovery was more partial than ROS inhibition and other previous treatments (Fig. 5d). These data suggest that autophagy is a key regulator for controlling the intracellular iron levels through ferritin degradation and the expression of TfR1 in response to erastin.

### Erastin-enhanced ROS leads to autophagy

**Fig. 6:**
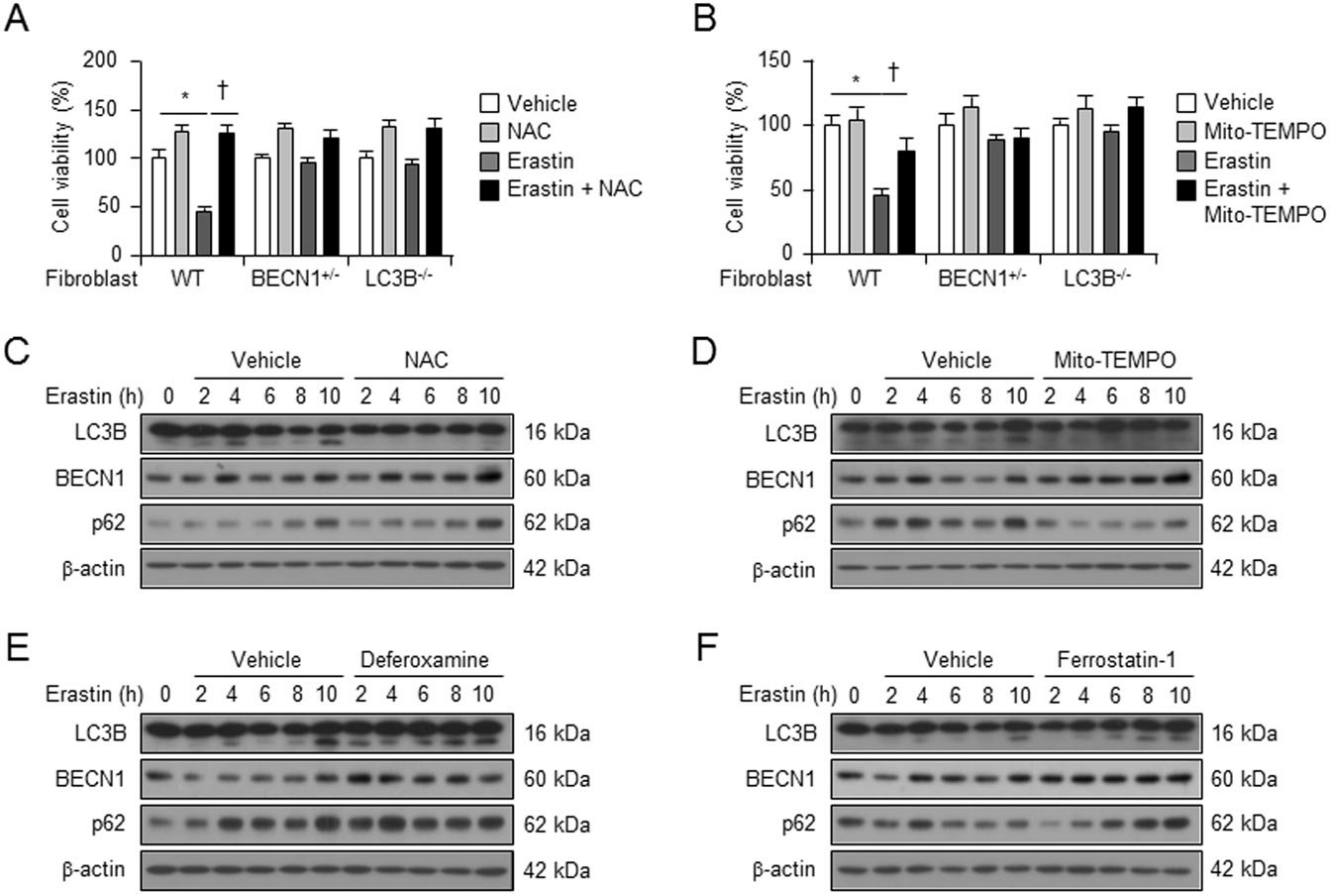
Autophagy was activated by erastin-mediated ROS in ferroptosis. The wild type (*n* = 9), BECN1^+/−^ (*n* = 9), or LC3B^−/−^ (*n* = 9) fibroblastic cells were treated with vehicle, NAC (1 mM), Mito-TEMPO (100 μM), erastin (5 μM), or erastin plus NAC or mito-TEMPO (**a**, **b**). Cell viability was analyzed 24 h after treatment. All experiments were performed at least three independent times. All data shown are the mean ± SD. *P *<* 0.05 indicates significant decrease compared with vehicle; ^†^*P* *<* 0.05 indicates significant increase compared with erastin alone. The wild-type fibroblastic cells were treated with vehicle or erastin (5 μM) in the absence or presence of ROS scavenger, NAC (**c**) or mito-TEMPO (**d**), iron chelator, deferoxamine (DFO) (**e**), or ferroptosis inhibitor, ferrostatin-1 (**f**). Total protein was harvested and the protein levels of LC3B, BECN1, and p62 were analyzed using western blot; β-actin was used as an internal loading control. These experiments were performed as three individual experiments and representative data is shown here

To confirm whether autophagy-induced ROS production is involved in erastin-induced cell death, we examined the impact of the antioxidant N-acetylcysteine (NAC) or the mitochondria-specific antioxidant mito-TEMPO on erastin-induced cell death in wild type, BECN1^+/−^, and LC3B^−/−^ fibroblastic cells. Pre-treatment with NAC or mito-TEMPO significantly recovered cell viability in wild-type fibroblastic cells (Fig. 6a, b). However, pre-treatment with NAC or mito-TEMPO had no significant effects on erastin-induced cell death in BECN1^+/−^ and LC3B^−/−^ fibroblastic cells (Fig. 6a, b). To further investigate whether erastin-induced ROS triggered autophagy, we treated wild-type fibroblastic cells with vehicle, erastin (5 μM) or erastin plus ROS inhibitors (NAC and mito-TEMPO), iron chelator (deforoxamine, DFO), or inhibitor for lipid peroxidation (ferrostatin-1) and harvested total protein 2, 4, 6, 8, or 10 h after cell treatment. Erastin treatment increased the levels of LC3B-II (lower band of LC3B) protein, while treatment with ROS inhibitors (NAC and mito-TEMPO) blocked autophagy in wild-type fibroblastic cells (Fig. 6c, d). However, erastin-induced autophagy was not blocked in the presence of iron chelator (deferoxamine, DFO) or inhibitor for lipid peroxidation (ferrostatin-1) (Fig. 6e, f). These data suggest that erastin-induced ROS triggers autophagy and results in increasing intracellular iron.

## Discussion

Ferroptosis is characterized as a type of programmed cell death that results from iron-dependent lipid peroxidation and is different from other types of cell death, especially autophagy^21^. In this study, we found that a ferroptotic inducer, erastin, enhanced the amount of LC3B-II in wild-type fibroblastic cells. Furthermore, viability of autophagy-deficient LC3B^−/−^ fibroblastic cells increased in the presence of erastin, compared with that of wild-type cells. Autophagy is a cellular catabolic degradation response to starvation or stress whereby cellular proteins, organelles and cytoplasm are engulfed, digested and recycled to sustain cellular metabolism^22^. Autophagy pathway is also used for the elimination of pathogens and for the engulfment of apoptotic cells^23,24^. However, the effect of these events on ferroptosis is not well understood. Although recent publications report a role of autophagy in ferroptotic cell death in breast cancer cells and wild-type mouse embryonic fibroblasts, their observations were contradictory^12–14,25^. Among the initial publications, Jiang’s group demonstrated that erastin-enhanced LC3 conversion in mouse embryonic fibroblasts (MEFs) and HT1080 cells and erastin-induced cell death were inhibited in the presence of autophagy inhibitors, suggesting that ferroptosis is an autophagic cell death process^13^. On the other hand, Gibson’s group insisted that the autophagy-induced cell death during ferroptosis occurred independently in breast cancer cells^12^ and Hou’s group also suggested that autophagy promotes ferroptosis by degradation of ferritin^14^. In their study, siramesine, a lysosome disruptor, and lapatinib, a dual tyrosine kinase inhibitor, initially induced ferroptotic cell death, but switched to autophagic cell death later on in breast cancer cells. Different inducers and cell types may induce different cellular mechanisms for ferroptotic cell death. However, in our study, erastin-induced ferroptotic cell death was clearly inhibited in autophagy-deficient BECN^+/−^ and LC3B^−/−^ fibroblastic cells (Fig. 1). This phenomena was confirmed by inhibition of erastin-induced ferroptotic cell death in the presence of autophagy inhibitors in wild type, BECN^+/−^, or LC3B^−/−^ fibroblastic cells (Fig. 3e). Our data strongly support the initial theory that ferroptosis is as an autophagic cell death process. Furthermore, we verified that erastin-increased reactive oxygen species (ROS) induced LC3B conversion and activated autophagy (Fig. 6).

With respect to the role of autophagy in ferroptosis, they demonstrated that autophagy regulates ferroptosis by regulating cellular iron homeostasis and cellular ROS generation^13^. Iron is a requisite metal in almost all biological systems. However, the levels of iron in the cell need to be tightly regulated, as an excess of iron can have damaging effects due to the generation of ROS^26^. Especially, autophagy can lead to the degradation of cellular iron stock protein ferritin and thus cause an increase of cellular labile iron levels, via NCOA4-mediated autophagy pathway, termed as ferritinophagy. High levels of cellular labile iron ensure rapid accumulation of cellular ROS, which is essential for ferroptosis^14^. Our data showed that the levels of intracellular iron were less in the autophagy-deficient cells compared with wild-type fibroblastic cells (Fig. 5). Consistently, the heavy subunit of ferritin, ferritin heavy chain 1 (FTH1), was degraded by erastin in wild-type cells, but not in autophagy-deficient cells, or in wild-type cells in presence of the autophagy inhibitor, 3MA (Fig. 5b, c), indicating that autophagy regulates the intracellular iron levels during erastin-induced ferroptotic process. Transferrin receptor is a carrier protein for transferrin, an iron-binding plasma protein, thus controlling the level of intracellular iron levels^20^. Interestingly, the protein levels of transferrin receptor (TfR1) were enhanced by erastin in wild-type cells, but not in autophagy-deficient cells and autophagy inhibitor-treated wild-type cells (Fig. 5b, c). Thus, our results demonstrate that autophagy regulates intracellular iron levels through ferritin degradation and transferrin receptor induction. Although autophagy has been reported during ferroptosis, the mediator of autophagy induction was not known. Gibson’s group suggested that prolonged iron-mediated ROS generation can induce autophagy^12^. However, our data showed that ROS inhibitors, NAC and mito-TEMPO, decreased erastin-induced autophagy, although the iron chelator, DFO, or lipid peroxidation inhibitor, ferrostatin-1 could not (Fig. 6). This indicates that iron-independent ROS is involved in induction of erastin-mediated autophagy.

## Materials and methods

### Reagents

Primary human dermal neonatal fibroblast (HDFn) and foreskin adult fibroblast (NuFF) cells were purchased from ATCC (Manassas, VA, USA), and cultured in DMEM medium (Gibco, Waltham, MA, USA) supplemented with 10% fetal bovine serum (Gibco, Waltham, MA, USA) and 1X Antibiotic-Antimycotic (100 U/mL penicillin, 100 μg/mL streptomycin, Fungizone^®^ 0.25 μg/mL, Gibco, Waltham, MA, USA) at 37 °C in a humidified incubator with 5% CO_2_. Mito-TEMPO was purchased from Enzo Life Sciences (Farmingdale, NY, USA). Erastin, 3-MA, chloroquine, deferipron, and NAC were from Sigma-aldrich (St. Louis, MO, USA). The following antibodies were purchased by Santa Cruz Biotechnology (Santa Cruz, CA, USA): anti-APG5 (sc-33210), anti-BECN1 (sc-11427), anti-LC3B (L7543), and anti-β-actin (sc-81178).

### Preparation of primary fibroblast from mouse lung

Primary pulmonary fibroblasts were isolated from LC3B^−/−^, BECN1^+/−^ mice and wild-type littermates, as previously described^24^. The lung tissue was soaked in a 1% antibiotic-antimycotic PBS (Invitrogen, Carlsbad, CA) twice for 20 min. The tissue was minced into 1-mm pieces, which were plated onto 100 mm dishes (30–40 pieces per plate). Fetal bovine serum was added dropwise over each tissue piece, and then the plates were incubated for 4 h at 37 °C. Then 2 mL of Dulbecco’s modified Eagle’s medium containing 10% fetal bovine serum and antibiotics was added to each plate. The plates were restored to the incubator and monitored every day until the fibroblasts reached confluence.

### Cell viability assay and propidium iodide (PI) staining assay

WST-1 assay was performed according to the manufacturer’s instructions (Roche, Indianapolis, IN, USA) with 10 μL of WST-1 reagent was added to each well of a 96-well plate (1 × 10^3^ cells/well). After 1 h of incubation using CO_2_ incubator, the conversion of WST-1 reagent into chromogenic formazan was monitored with a spectrophotometer. On day 1 after plating, cells were treated with various doses (2, 5, and 10 µM) and times (8, 16, and 24 h) of erastin (Sigma, St. Louis, MO), respectively. PI staining assay was analyzed using flow cytometry. Fibroblast cells were seeded in 6-well plates at a density of 3 × 10^5^ cells/well. For drug treatment experiments, we treated fibroblast with erastin 5 μM and/or rapamycin (5 μM), 3-methylaldehyde (2 mM). After treat for 11 h, the cells were harvested, washed with phosphate-buffered saline (PBS), and stained using the propidium iodide (PI) cell death Detection Kit (Becton Dickinson, San Jose, CA, USA). Measurements were performed on a FACSCalibur (Becton Dickinson, San Jose, CA, USA) flow cytometer. Fluorescence of each probe was measured using FlowJo software program. The mean percentages ± SD of positive cells per total cells are shown in plots.

### siRNA and transient transfection

Mouse LC3B, BECN1, and control scramble-siRNA were from Bioneer Corporation (Deajeon, South Korea). Fibroblastic cells were transfected with siRNA at 0.1 μM using FuGENE6 transfection reagent (Roche, Indianapolis, IN, USA)) according to the manufacturer’s instructions. The sequences of siRNA were as follows: mouse lc3b (sence sequence: GUG GUU GUC AAG UGG UAG A (dTdT), antisence sequence: UCU ACC ACU UGA CAA CCA C (dTdT)); mouse - becn1 (sence sequence: GAC CAU GCA AUG GUA GC U (dTdT), antisence sequence: AAG CUA CCA UUG CAU GGU C (dTdT)).

### Immunoblot analysis

All samples were lysed with radioimmunoprecipitation assay (RIPA) lysis buffer (50 mM Tris-HCl (pH 7.4), 150 mM NaCl, 1% NP40, 0.25% sodium deoxycholate, 1 mM phenylmethylsulfonylfluoride (PMSF), 1 mM sodium orthovanadate, 1× sigma protease inhibitor cocktail) in ice for 30 min. Lysates were then quantified via Pierce^®^ BCA Protein Assay Kit (Thermo scientific, Rockford, IL, USA). In total, 10–50 μg of protein was separated by 10–15% SDS-PAGE and then transferred onto Immobilon^®^-P membrane (Millipore, Billerica, MA, USA). The membranes were blocked with 5% skim milk/TBS-T for 1 h and incubated with primary antibodies for overnight at 4 °C. After washed three times, the membranes were incubated with HRP-conjugated secondary antibodies for 40 min at room temperature. After 1.5 h washing, protein bands were visualized using Chemiluminescent HRP Substrate (Millipore, Billerica, MA, USA).

### Iron assay

Levels of total iron were analyzed in wild type, BECN1^+/−^, and LC3B^−/−^ fibroblastic cells using the kit as described^26^. An iron assay kit (Abcam, Cambridge, MA) was subsequently used to quantify total iron in the cell lysates according to manufacturer’s instructions. Briefly, cells were harvested from a confluent T75 for each analysis. Cells can be lysed in 4 volume of iron assay buffer, centrifuge at 16,000 × *g* for 10 min to remove insoluble materials. Five microliters of iron reducer were added into 50 µl samples for total iron (Fe^3+^ plus Fe^2+^) assay. Next, 100 μL iron probe solution was added into samples and incubated at 25 °C for 60 min protected from light. Spectrophotometry was used to detect absorbance at 593 nm wavelength.

### Assessment of cytosolic ROS and lipid peroxidation

Cells were seeded at 3 × 10^5^ cells per well in 6-well plates. Next day, cells were treated with erastin (10 μM) and/or chloroquine (5 μM), 3-methylaldehyde (10 μM) for 8 h. After 8 h, cells were incubated with 2 mM CellROX® Deep Red (cytosolic ROS) or 2 μM C11-BODIPY581/591 (lipid peroxidation) (Invitrogen, Life Technologies, Grand Island, NY) for 30 min at 37 °C in the dark. After 30 min of loading, unincorporated dye was removed by washings with 2% FBS containing PBS. Samples were then centrifuged at 1000 rpm for 3 min and the pellets were resuspended in 500 μL of 2% FBS containing PBS Measurements were performed on a FACSCalibur (Becton Dickinson, San Jose, CA, USA) flow cytometer. Fluorescence of each probe was measured using FlowJo software program. The mean percentages ± SD of positive cells per total cells are shown in plots.

### Statistical analysis

All results were confirmed in at least three independent experiments; data from one representative experiment are shown. Quantitative data are shown as means ± standard deviation and significance of statistical analysis was determined with two-tailed, unpaired Student’s *t*-test. *P*-values < 0.05 were considered significant.

## Supplementary information

CDDIS-18-2909R Supplemental Figure legends

Supplementary Figure 1

Supplementary Figure 2
