# Supplementary material for: ROS-mediated autophagy increases intracellular iron levels and ferroptosis by ferritin and transferrin receptor regulation
Source: Cell Death Dis. 2019 Oct 28;10(11):822. doi: 10.1038/s41419-019-2064-5 (PMC6817894; doi:10.1038/s41419-019-2064-5)
Supplement: Supplementary file 1 — CDDIS-18-2909R Supplemental Figure legends [file 41419_2019_2064_MOESM1_ESM.docx]

**ROS-mediated autophagy increases intracellular iron levels and ferroptosis by ferritin and transferrin receptor regulation**

Eunhee Park and Su Wol Chung

School of Biological Sciences, College of Natural Sciences, University of Ulsan, 93 Daehak-ro, Nam-gu, Ulsan 44610, South Korea

Running title: Role of autophagy in ferroptosis

Address correspondence to:

Su Wol Chung, Ph.D., School of Biological Sciences, College of Natural Sciences,

University of Ulsan, 93 Daehak-ro, Nam-gu, Ulsan 680-749, South Korea

Tel: 82-52-259-1641; Fax: 82-52-259-1694; E-mail:swchung@ulsan.ac.kr

**Supplementary Figures legends**

**Supplementary Figure 1. Rapamycin-induced autophagy was impaired in BECN**^+/–^ **and LC3**^–/–^ **fibroblastic cells.** Vehicle or rapamycin was treated in BECN^+/+^, BECN^+/–^, LC3B^+/+^, and LC3B^–/–^ fibroblastic cells at various time points. LC3B, BECN1, and p62 were assessed by Western blot analysis. β-actin was used as controls for normalization.

**Supplementary Figure 2. Erastin-induced ferroptosis was accelerated in the presence of rapamycin-induced autophagy in wild type fibroblastic cells.** Wild type fibroblast cells were treated with erastin in the presence of rapamycin (**A**) or 3-methylaldehyde (**B**) and stained with propidium iodide (PI) to check cell death. PI stained cells were analyzed using a flow cytometer. Values are mean ± SD, n=8. **P <* 0.05 indicates significant increase compared with vehicle or erastin alone. ^†^P < 0.05 indicates significant increase compared with erastin alone.
