# Supplementary figures and images for: ROS-mediated autophagy increases intracellular iron levels and ferroptosis by ferritin and transferrin receptor regulation
Source: Cell Death Dis. 2019 Oct 28;10(11):822. doi: 10.1038/s41419-019-2064-5 (PMC6817894; doi:10.1038/s41419-019-2064-5)

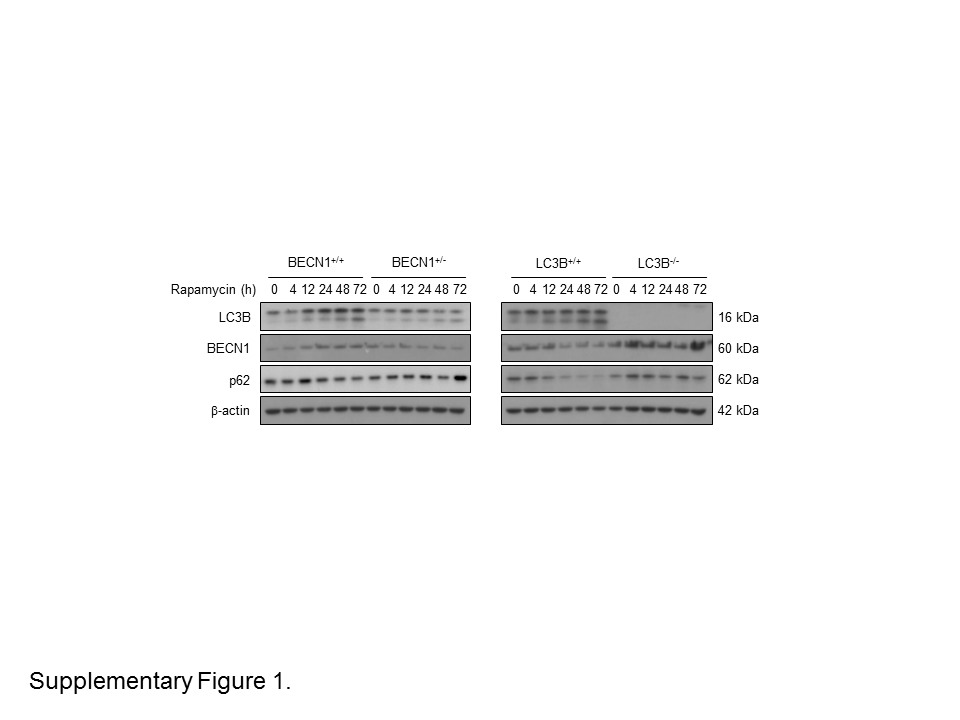

Supplement: Supplementary file 2 — Supplementary Figure 1 [file 41419_2019_2064_MOESM2_ESM.tif]

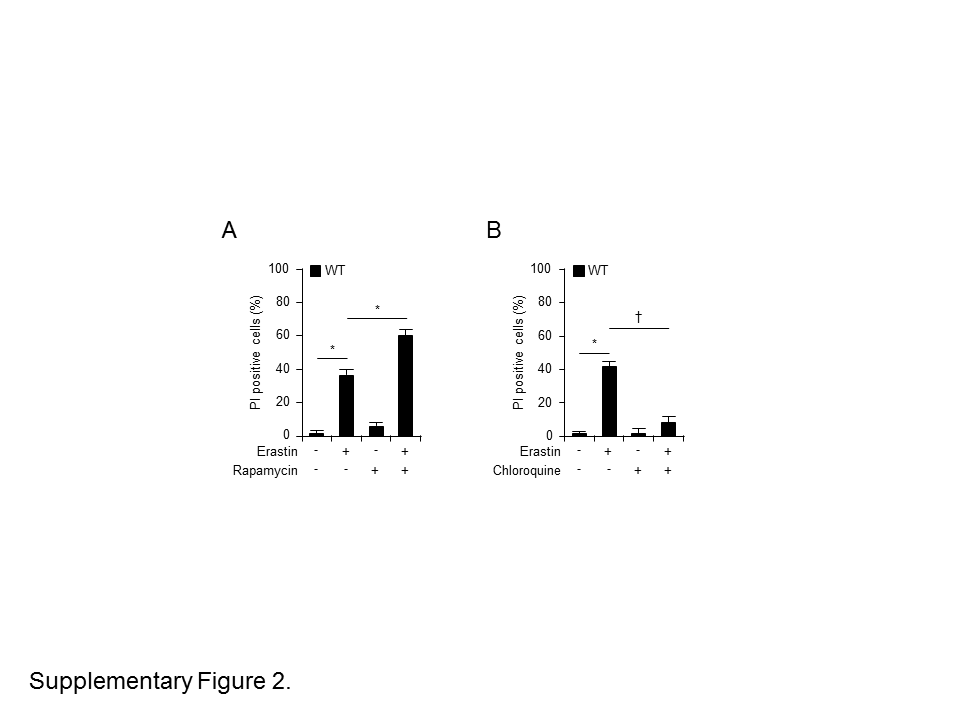

Supplement: Supplementary file 3 — Supplementary Figure 2 [file 41419_2019_2064_MOESM3_ESM.tif]
